# Cytotoxic Efficiency of Human CD8^+^ T Cell Memory Subtypes

**DOI:** 10.3389/fimmu.2022.838484

**Published:** 2022-04-13

**Authors:** Arne Knörck, Gertrud Schäfer, Dalia Alansary, Josephine Richter, Lorenz Thurner, Markus Hoth, Eva C. Schwarz

**Affiliations:** ^1^ Biophysics, Center for Integrative Physiology and Molecular Medicine, School of Medicine, Saarland University, Homburg, Germany; ^2^ Molecular Biophysics, Center for Integrative Physiology and Molecular Medicine, School of Medicine, Saarland University, Homburg, Germany; ^3^ Internal Medicine I, School of Medicine, Saarland University, Homburg, Germany

**Keywords:** cytotoxic T lymphocytes, CTL, memory CD8^+^ T cells, SEA (staphylococcal enterotoxin A), killing mechanism, perforin, CD8^+^ T cell subtypes

## Abstract

Immunological memory is important to protect humans against recurring diseases. Memory CD8^+^ T cells are required for quick expansion into effector cells but also provide immediate cytotoxicity against their targets. Whereas many functions of the two main cytotoxic subtypes, effector memory CD8^+^ T cells (T_EM_) and central memory CD8^+^ T cells (T_CM_), are well defined, single T_EM_ and T_CM_ cell cytotoxicity has not been quantified. To quantify cytotoxic efficiency of T_EM_ and T_CM_, we developed a FRET-based single cell fluorescent assay with NALM6 target cells which allows analysis of target cell apoptosis, secondary necrosis following apoptosis, and primary necrosis after T_EM_- or T_CM_-target cell contact. Both, single cell and population cytotoxicity assays reveal a higher cytotoxic efficiency of T_EM_ compared to T_CM_, as quantified by target cell apoptosis and secondary necrosis. Perforin, granzyme B, FasL, but not TRAIL expression are higher in T_EM_ compared to T_CM_. Higher perforin levels (likely in combination with higher granzyme levels) mediate higher cytotoxic efficiency of T_EM_ compared to T_CM_. Both, T_EM_ and T_CM_ need the same time to find their targets, however contact time between CTL and target, time to induce apoptosis, and time to induce secondary necrosis are all shorter for T_EM_. In addition, immune synapse formation in T_EM_ appears to be slightly more efficient than in T_CM_. Defining and quantifying single T_EM_ and T_CM_ cytotoxicity and the respective mechanisms is important to optimize future subset-based immune therapies.

## Introduction

Cellular cytotoxicity mediated by activated CD8^+^ cytotoxic T cells (CTL) is a crucial function of the adaptive immune system to efficiently fight cancer or viral infections. CTL kill virus-infected cells or tumor cells by various effector mechanisms. Directed release of lytic granules containing perforin and granzymes or death receptor engagement mediated by FasL or TRAIL are the most prominent mechanisms (1–5).

CTL are central to modern cancer immunotherapy. In the ideal scenario, CTL specifically detect MHCI-presented antigens on the surface of tumor cells and finally kill these targets (6, 7). The outcome of therapies using genetically modified T cell populations is influenced by central or peripheral tolerance, tumor- or virus-associated immune suppression, functional defects of the MHCI-machinery mitigating presentation of tumor neo- or viral antigens, or the occurrence of severe side-effects like the cytokine release syndrome (CRS) or neurotoxicity (NT) (8, 9). To date, the majority of T cell therapies rely not on defined T cell subsets but the entire T cell population (10). However, recent data support the idea that pre-selection of T cell subsets might help to optimize further treatments (11–13). Unfortunately, it is problematic to achieve this because patients are often already very sick with severe lymphopenia. Nevertheless, a detailed understanding CTL subset cytotoxicity is needed to optimize future cellular T cell therapies.

T cell memory subsets, T_EM_ or T_CM_, differentiate during an immune response and provide long term immunity against recurring viral or tumour antigens. The different subpopulations are usually defined by surface expression of CCR7/CD62L and CD45RO/RA (14, 15). Whereas T_EM_ likely respond immediately to a recurring antigen, T_CM_ reside in the secondary lymphatic tissues keeping a comparably high proliferative capacity to develop a new population of effector cells after a second conjugation to the same antigen (16, 17). Although T_EM_ are expected to have a higher cytotoxic potential compared to T_CM_, detailed functional information about the orchestration of different killing mechanisms and the killing machinery, which is used by distinct subtypes, is still missing.

In this study, we developed a single cell kinetic assay to quantify cancer cell apoptosis and necrosis following T_EM_ or T_CM_ contact. We show that T_EM_ and T_CM_ utilized both, perforin and death receptor mediated mechanisms to kill target cells. While perforin/granzyme-mediated induction of apoptosis is the most prevalent mechanism for both subsets, T_EM_ showed an increased cytotoxic potential which is not only caused by higher perforin expression but we also find a shortened time to establish the first killer-target cell contact with a subsequent successful killing.

## Results

### Subtype Distribution of SEA-Stimulated CD8^+^ T Cells (SEA-CTL)

**Figure 1:**
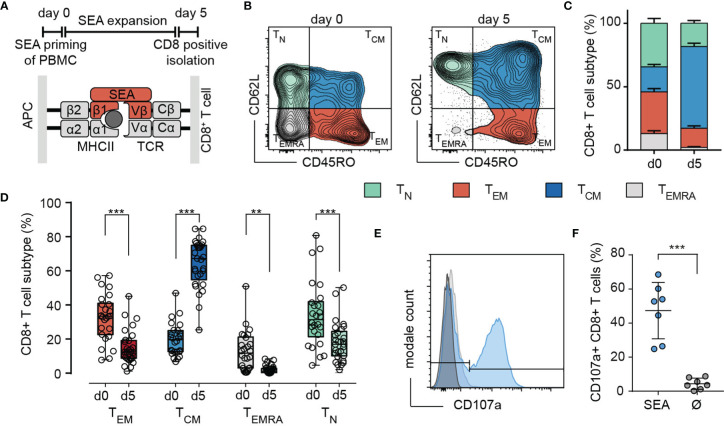
SEA stimulation induces differentiation mainly into a T_CM_ subtype. **(A)** Scheme of the 5-day stimulation with SEA (Staphylococcal enterotoxin A) and the mechanism of CTL-target cell crosslinking by the SEA molecule. **(B)** Representative example for gating of (CD3^+^/CD8^+^) CTL into T_N_ (CD62L^+^/CD45RO^-^), T_CM_ (CD62L^+^/CD45RO^+^), T_EM_ (CD62L^-^/CD45RO^+^) and T_EMRA_ (CD62L^-^/CD45RO^-^) on day 0 and day 5 of SEA-stimulation. **(C, D)** n= 26-29 donors. **(C)** Quantification of subtype distribution at day 0 and day 5. Data are presented as mean+SEM. **(D)** Compared frequencies of T_N_, T_CM_, T_EM_ and T_EMRA_ on day 0 and day 5. Box and Whisker plots show min, max, median, and 25-75% interquartile range. Statistical analysis was done by a two-way ANOVA. **(E, F)** Detection of SEA-dependent degranulation by CD107a surface expression of SEA-CTL after co-incubation with SEA-pulsed Raji cells. **(E)** Degranulation of cells from a representative donor (-SEA: grey, +SEA: blue. **F**) Quantification of **(E)** with SEA-loaded (SEA) and control (no SEA, Ø) target cells. Data are shown as mean ± SD of 7 donors. **p<0.01; ***p<0.001.

Upon activation, cytotoxic T-lymphocytes (CTL) differentiate into various subsets to execute their cytotoxic function. In this study, we used a widely accepted protocol for activation of T cells by staphylococcal enterotoxin A (SEA). PMBC were isolated from leukoreduction system (LRS) chambers and pulsed with SEA. CTL were positively isolated after the expansion of the mixed PBMC population for 5 days (**Figure 1A**).

CD8^+^ naïve and memory T cell subsets were identified by the surface molecules CD62L or CCR7 and CD45 isoforms RA and RO according to (14). **Figure 1B** illustrates that upon SEA stimulation, the CD8^+^ population was shifted towards a T_CM_ phenotype. The frequency of the T_CM_ subset increased from 19.8 ± 9.5% on day 0 to be the predominant subset (64 ± 5%) on day 5 of SEA-stimulation. Frequencies of T_EM_ and T_N_ were decreased by roughly 50% (T_EM_ 53.9%, T_N_ 46.7%) to 15.1% and 18.3%, while most of the T_EMRA_ cells disappeared during the expansion period (**Figure 1C**). Healthy human donors show a huge variation in subtype distribution but T_CM_ was always the prominent subtype after SEA stimulation whereas T_EM_ and T_N_ decreased, and T_EMRA_ was almost absent (**Figure 1D**). To test the SEA reactivity of stimulated CTL (SEA-CTL), we analyzed the degranulation after co-incubation with SEA-pulsed target cells by detection of surface-located CD107a (**Figures 1E, F**). On average, 47.3 ± 16.5% of SEA-CTL degranulated in case target cells were loaded with SEA compared to 4.4 ± 3.3% in case target cells were not loaded with SEA (**Figure 1F**).

**Figure 2:**
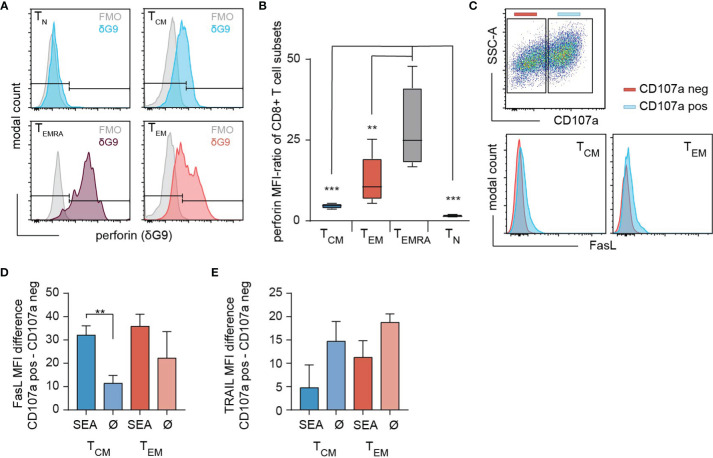
Expression of death mediators in SEA-CTL. **(A)** Content of intracellular perforin stores analyzed in SEA-CTL (bead-isolated SEA-stimulated CD8^+^ population) subsets defined by CCR7 and CD45RO (clone δG9 for perforin in lytic granules) shown for a representative donor. **(B)** Quantification of subset specific perforin in lytic granules (clone δG9) by MFI-Ratio (MFI (perforin^+^ population)/MFI (FMO)) shown for 5 donors. Box and Whisker plots show min, max, median, and 25-75% interquartile range. Statistical analysis was done by a two-way ANOVA. **(C)** CTL-SEA were co-incubated with 3x10^5^ SEA-pulsed NALM6 at an E:T ratio of 2:1 for 4h in presence of CD107a antibody. Cells were collected and stained with CD45RA, CCR7, CD95L and TRAIL (CD253) antibodies. CD107a positive (CD107a pos, blue) and CD107a negative (CD107a neg, red) cells were gated for each subset (T_CM_ and T_EM_) and FasL expression was analyzed. Quantification of FasL **(D)** or TRAIL **(E)** expression on degranulating SEA-CTL (SEA) or control cells (without the addition of SEA, Ø) cells in T_CM_ and T_EM_ subpopulations. Data are shown as mean+SD, n=3 donors. **p<0.01; ***p<0.001.

To assess the cytotoxic potential of each individual subset, we determined the perforin expression in T_N_, T_CM_, T_EM_ and T_EMRA_ (**Figure 2A**). The antibody clone δG9v identifies granule-associated perforin ready to lyse the target cell (18). The content of perforin was higher in T_EM_ compared to T_CM_. Although the frequencies of perforin expressing cells are comparable among T_EM_ (70.17 ± 12%) and T_CM_ (65.06 ± 8.9%) (**Figure 2A**), the analysis of the perforin MFI-ratios reveals that T_EM_ express 2.8 times more granular perforin than T_CM_ (**Figure 2B**). T_EMRA_ express a significantly increased amount of perforin (**Figure 2B**) but the subpopulation is almost absent after SEA-stimulation (2.14 ± 2.4% of all cells). The death receptors FasL and TRAIL are also expressed in T_CM_ and T_EM_. However, only FasL (**Figures 2C, D**) but not TRAIL (**Figure 2E**) were moderately upregulated after SEA-loaded target cell contact. In conclusion, after expansion by SEA, T_EM_ and T_CM_ are the main cytotoxic-competent subpopulations.

### Subset Specific Cytotoxic Potential of T_EM_ and T_CM_ Subpopulations

**Figure 3:**
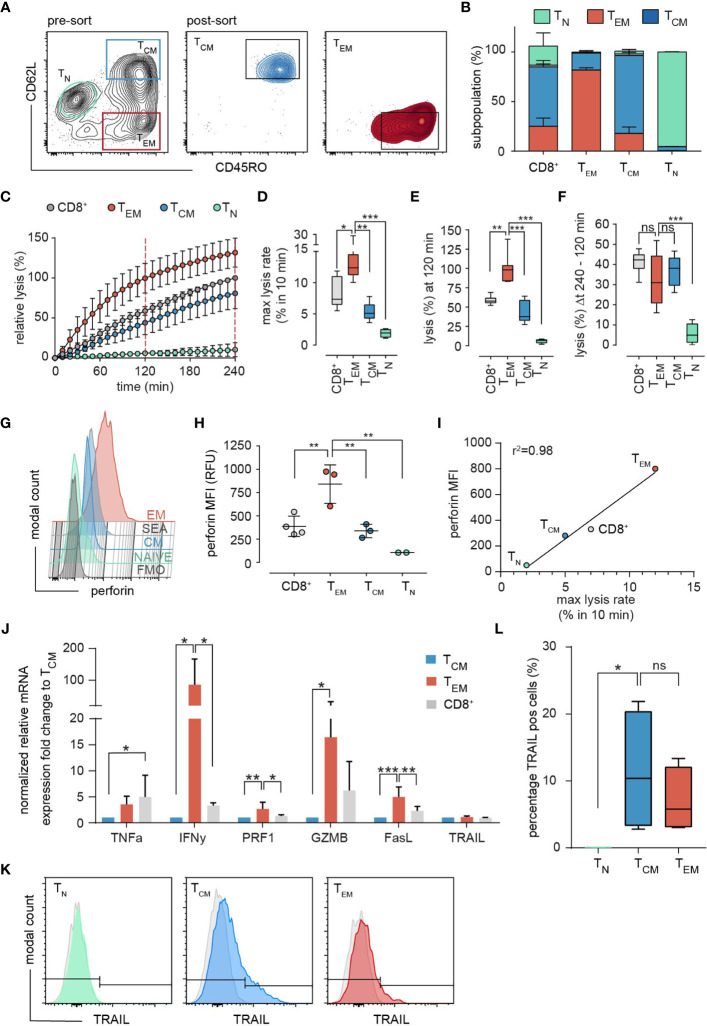
Subset-specific cytotoxicity of sorted T_EM_ and T_CM_. **(A)** Representative example of the sorting strategy. After exclusion of doublets and dead cells by light scatter parameters, the SEA-CTL (CD8^+^) population was separated and sorted into T_EM_ and T_CM_ cells by the surface marker CD62L and CD45RO. 24h after sorting cells were re-analyzed by a staining of CD45RA and CCR7. The subset composition of sorted samples is shown as stacked bar graphs **(B)**, (T_EM_, red; T_CM_, blue; T_N_, green). **(C)** Cytotoxicity of sorted T_EM_, T_CM_, T_N_ and of the unsorted SEA-CTL population (CD8^+^) was analyzed by a calcein-based real-time killing assay. **(D-F)** To quantify the killing efficiency, the maximal lysis rate (maximal target cell lysis within a 10 min interval), the target lysis between 0 - 120 min **(E)** and 120 min - 240 min **(F)** were analyzed (number of donors, SEA-CTL (CD8^+^) n=9, T_EM_ n=8, T_CM_ n=7, T_N_ n=4 donors). Box and whisker plots in D-F show min, max, median, and 25-75% interquartile range. Statistical analysis was done by a one-way ANOVA. **(G)** Perforin expression in sorted T_EM_, T_CM_, T_N_ and in the unsorted SEA-CTL (CD8^+^) population of a representative donor analyzed by flow cytometry. **(H)** Quantification of intracellular perforin by MFI. SEA-CTL (CD8^+^) n=4, T_EM_ n=3, T_CM_ n=3, T_N_ n=2 donors. Scatter plots show mean ± SD, statistical analysis was done by a one-way ANOVA. **(I)** MFI of intracellular perforin **(H)** plotted against maximal lysis rates **(D)**. **(J)** Expression of various effector molecules, TNFa, IFNy, PRF1, GZMB, FASL and TRAIL genes was analyzed on mRNA level by RT-qPCR, 48h after sorting. Relative mRNA expression is shown as fold change normalized to the T_CM_ population. T_EM_ and T_CM_ n=4, SEA-CTL (CD8^+^) n=2 donors. Bar graphs show means ± SD. **(K)** Expression of TRAIL in T_N,_ T_CM_ and T_EM_ subsets analyzed by flow cytometry. **(L)** Percentage of TRAIL positive cells was quantified as a box and whisker plot. N = 4 donors. Statistical analysis was done by a Kruskal-Wallis-Test. *p<0.05; **p<0.01; ***p<0.001; ns, no significant difference.

To determine the cytotoxic potential of individual memory subsets we sorted bead-isolated SEA-stimulated CD8^+^ population (SEA-CTL) into CD62L^+^/CD45RO^+^ T_CM_ and CD62L^-^/CD45RO^+^ T_EM_ subpopulations (**Figure 3A**, pre- and post-sort). We also sorted CD62L^+^/CD45RO^-^ T_N_ from SEA-CTL of 4 different donors. We used these T_N_ as an internal negative control, although the cells cannot be considered as truly naïve since they were in the environment of SEA stimulation for 4 days. However, in terms of their surface markers, they are considered T_N_. 24h after subpopulation sorting, we re-analyzed the sorted T_EM_ or T_CM_ cells and found a purity of >80% (**Figure 3B**). At the same time, we analyzed the killing capacity of the different subpopulations in a time-resolved population killing assay (**Figure 3C**). This assay allows quantification of the cytotoxic potential at different time points and quantification of the maximum lysis rate which describes the maximal difference in target cell lysis between two measurement points (10 min). As expected, T_N_ did not kill target cells. The T_EM_ subpopulation displayed the highest cytotoxic efficiency (**Figure 3C**) as also quantified by the maximal lysis rate (**Figure 3D**) and the target cell lysis at 120 min (**Figure 3E**) compared to T_CM_, T_N_ and the entire SEA-CTL population as control. Interestingly, the change of target lysis was not different during the last 2 hours (between 120 min and 240 min) among T_EM_, T_CM_ and SEA-CTL (**Figure 3F**, also compare **Figure 3C**). Thus, the main difference between T_EM_ and T_CM_ regarding cytotoxic efficiency is attributed to the initial phase of the cytotoxicity assay (within the first 2 hours).

Since perforin-mediated exocytosis is known to be rapidly executed (19–22), and since we and others have shown that perforin determines the initial killing phase in CTL and NK cells (22, 23), we explored the perforin content in sorted subsets in parallel to the population killing assay to confirm the increased expression of granule-associated perforin (using antibody clone ΔG9), which we observed for T_EM_ before in in non-sorted subsets (**Figures 2A, B**). Again, we found a significant difference in perforin content of T_EM_ compared to T_CM_ cells (**Figures 3G, H**). The SEA-CTL (CD8^+^) control-population expressed slightly more perforin than T_CM_ but less than T_EM_, which is in good agreement with the subtype composition (T_CM_>T_EM_>T_N_). T_N_ sorted cells have almost no perforin (**Figure 3H**) in good accordance with the negligible killing efficiency shown before (**Figure 3C**). The mean fluorescence intensity of perforin correlated almost perfectly with the maximal lysis rate of the subtypes and the entire SEA-CTL (CD8^+^) population (**Figure 3I**, r^2^ = 0.98). Therefore, it is likely that the differences of initial lysis of target cells during the first 120 min are caused by differences in perforin content.

When analyzing various effector molecules important for killing mechanisms on mRNA level, we also found interferon-γ, granzyme B and FasL but not TNFα or TRAIL significantly enhanced in T_EM_ (**Figure 3J**). The same was true for the surface expression of TRAIL analyzed by flow cytometry (**Figures 3K, L**), suggesting that additional, possibly slower killing mechanisms could contribute to the enhanced T_EM_ mediated target cell lysis.

### Analysis of T_EM_ and T_CM_ Induced Target Cell Lysis at Single Cell Level Using the Apoptosis Sensor Casper-GR

So far, our results revealed that the differences in subset-specific cytotoxicity measured with the population killing assay were likely caused by perforin-mediated target cell lysis (**Figure 3C**), probably also involving granzyme B, which is expressed at a higher level in T_EM_ compared to T_CM_ **Figure 3J**). To detect potential other mechanisms involved in the differential cytotoxic efficiency of T_EM_ compared to T_CM_ like target cell contact times and/or functional immune synapse formation, single cell resolution is required. Additionally, population-mediated assays usually do not allow to discriminate between apoptotic or necrotic target cell death. Numerous tools to dissect the different mechanisms during target cell lysis have been developed in the past years (20–22, 24) but the diversity between different subtypes was not in the focus of this research. The recently introduced microscope-based assay (24) allows the detailed analysis and identification of apoptotic and necrotic lysis events on a single cell level by using the FRET-based apoptosis sensor Casper-GR (25). This assay was up to now only applied to natural killer (NK) cells (24). To establish and validate this assay for human CTL, we first tried to stably transfect the clonal B cell lymphoma target cell line Raji (used for the population killing assay, **Figure 3C**) with pCasper-GR. However, expression levels were highly variable compromising a detailed quantification. Therefore, we turned to the target cell line NALM6, a B cell line established from a patient with acute lymphoblastic leukemia (ALL) which can also be loaded with SEA.

To validate NALM6 and Raji cells as targets against each other, we compared CTL cytotoxicity in the population killing assay. Neither the kinetics of killing nor the maximal lysis rate were different (**Supplementary Figure 1**) indicating that human CTL eliminate both targets equally efficient. We also compared FasR and TRAIL receptor expression. Both, NALM6 and Raji cells, express FasR and TRAIL receptors R1-R3 but only very little (Raji) or no (NALM6) TRAIL-R4. While the overall expression of NALM6 target cells is slightly lower compared to Raji cells, the number of positive cells is comparable (**Supplementary Figure 1**).

**Figure 4:**
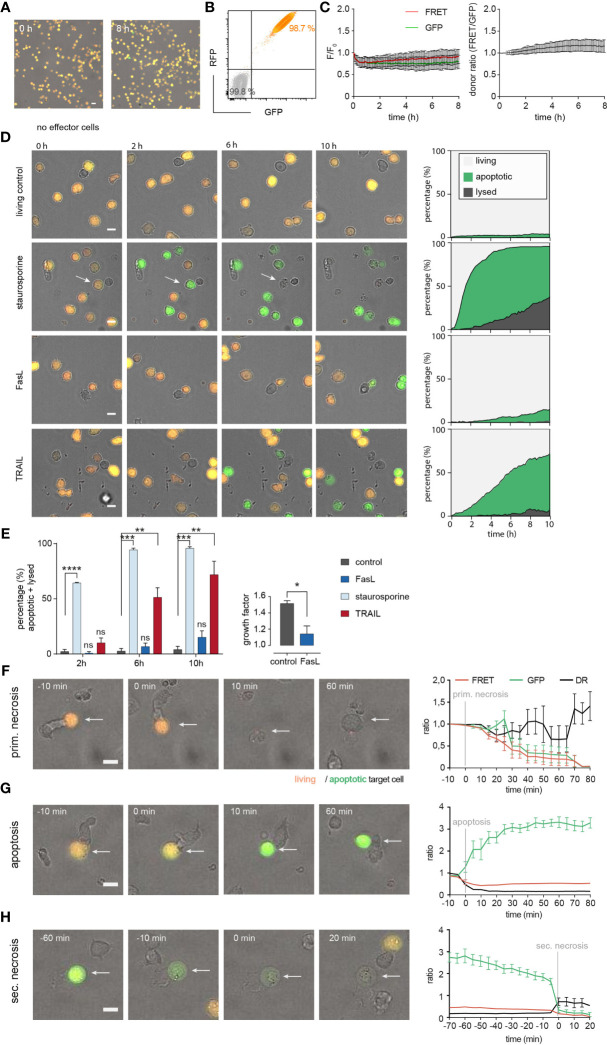
Establishing NALM6 pCasper at single cell level as target cells for SEA-CTL to distinguish between apoptotic and necrotic cell death. **(A)** Viability of NALM6 pCasper cells at 37° C, 5% CO2 for 8h. Viable cells are orange, apoptotic cells are green. **(B)** NALM6 cells were stably transfected with pCasper-GR. GFP and RFP fluorescence of NALM6 pCasper in comparison to non-transfected NALM6 cells (grey) detected by flow cytometry as dot blot. **(C)** Time-resolved averaged normalized GFP and FRET fluorescence (left) and FRET donor ratio (right) over 8h. **(D, E)** Detection of apoptosis induction by staurosporine, anti-Fas (Apo1-1) mimicking FasL or recombinant TRAIL. **(D)** Representative images and automated death plot analysis of NALM6 pCasper cells (control, n=426 cells) over 10h treated with 20 µM staurosporine (n=283 cells), 5 µg/ml APO1-1 (n=254 cells) or 5 µg/ml recombinant TRAIL (n=405 cells) are shown. **(E)** Quantification of the fraction of apoptotic and lysed cells and the effect of Apo1-1 treatment (p=0.037) on the growth factor (mean #cells (600 min)/#cells (0-60 min)), single donor, duplicates. Statistical analysis was done using a Friedman test. Specific combinations of substances were used to inhibit perforin and death receptor mediated cytotoxic mechanisms during the assay and by 2h of preincubation. **(F)** Representative lysis event showing a primary necrosis and time-resolved averaged normalized GFP and FRET fluorescence of five cells. **(G)** Representative lysis event showing an apoptosis and time-resolved averaged normalized GFP and FRET fluorescence of seven cells. **(H)** Representative lysis event showing a secondary necrosis following an apoptosis and time-resolved averaged normalized GFP and FRET fluorescence of seven cells. **(F-H)** SEA-CTL were used as effector cells. Scale bars are 10 µm. * p<0.05; ** p<0.01; ***p<0.001, ****p<0.0001; ns, no significant difference.

Thus, we were confident to use stably-transfected Casper-GR NALM6 cells (from now on named NALM6 pCasper) in the following single cell experiments as target cells. 98.7% of the NALM6 pCasper population was homogenously and strongly positive for GFP and RFP signals (**Figures 4A, B**). To monitor the viability of NALM6 pCasper under experimental conditions (37°C, 5% CO_2_) we recorded the GFP and FRET signals at 5 min intervals which remained stable over the complete 8 hours measurement period (**Figure 4C**). To test the functionality of the apoptosis sensor, staurosporine was applied to the targets (without using SEA-CTL effector cells), since staurosporine induces caspase-3 mediated apoptosis (26). In addition, we tested the induction of death receptor-mediated apoptosis by the anti-Fas antibody Apo1-1 mimicking FasL and by recombinant TRAIL to induce TRAIL-receptor mediated cell death over 10 hours. The effects of staurosporine, Apo1-1 and recombinant TRAIL are depicted in **Figure 4D** compared to untreated control. The quantitative analysis of target cell death is illustrated in a stacked diagram over time called death plots. These illustrate the kinetic distribution of lysed cells (dark grey), apoptotic (green) and living cells (light grey). After 10 hours, in comparison to the untreated living control, staurosporine and recombinant TRAIL significantly enhanced apoptotic cell deaths (95.7% ± 1.5% and 71.8% ± 12.3%, respectively) whereas Apo1-1 was relatively inefficient in inducing apoptotic cell death.

In contrast to staurosporine and TRAIL, the addition of Apo1-1 did not induce a significant increase in the proportional sum of apoptotic and lysed cells even after 10 hours compared with the control condition (**Figure 4E**, left panel). Interestingly, when we counted the number of Apo1-1 treated cells, the calculated growth factor was significantly decreased compared to untreated control cells meaning the anti-Fas antibody Apo1-1 reduced the proliferation of NALM6 pCasper (**Figure 4E**, right panel).

Next, we analyzed the modes of cell death occurring in NALM6 pCasper after contact with SEA-CTL. Target cell apoptosis and necrosis following CTL contact could be distinguished as previously established for NK cells (24). We confirmed that the assay allows to distinguish primary necrosis (**Figure 4F**), apoptosis (**Figure 4G**) and secondary necrosis (**Figure 4H**) of target cell induced after SEA-CTL contact. Primary necrosis of NALM6 pCasper target cells induced by SEA-CTL is characterized by the parallel loss of the GFP- and FRET fluorescence signals accompanied by morphological changes (**Figure 4F**). The induction of caspase-mediated apoptosis following SEA-CTL contact was identified by an increase of the GFP-fluorescence signal and a decrease of the FRET-donor ratio (DR) (**Figure 4G**). This is expected since GFP- and RFP-molecules of the pCasper FRET-construct are linked by the caspase recognition sequence DEVD which is cleaved by different caspases after induction of apoptosis (caspase-3, -7 and others). Secondary necrosis following initial apoptosis is characterized by a sudden drop of GFP- and FRET-fluorescence signals accompanied by an increase of the cell volume typically for necrotic cell death (**Figure 4H**). In summary, NALM6 pCasper are suitable targets to study different modes of CTL-mediated target cell death.

### Single Cell Level T_EM_ and T_CM_ Analyses Reveal Distinct Cytotoxic Efficiencies

The population real-time killing assay revealed clear differences between T_EM_ and T_CM_ cytotoxic efficiencies (**Figure 3C**). However, it does not allow any conclusions on death modes. Therefore, we used the single cell killing assay to compare T_EM_ and T_CM_ to obtain insights into distinct target cell death modes.

**Figure 5:**
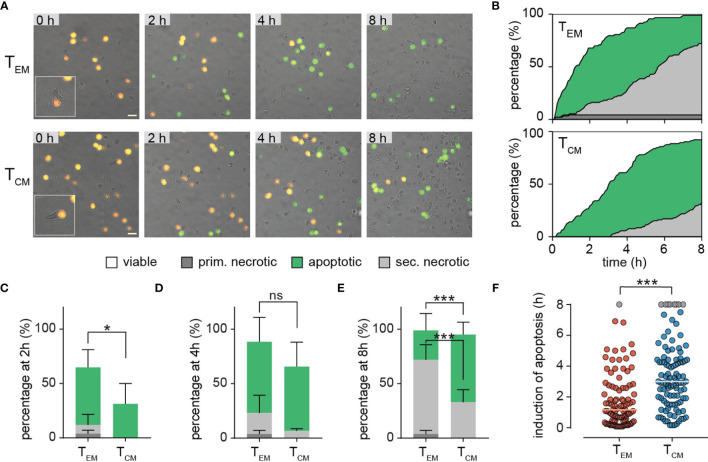
Single cell level T_EM_ and T_CM_ analyses reveal distinct cytotoxic efficiencies. **(A, B)** Sorted T_EM_ and T_CM_ were co-incubated with SEA-pulsed NALM6 pCasper cells at an E:T ratio of 2:1 for 8h to analyze cytotoxicity on single cell level. **(A)** Representative image section of T_EM_ or T_CM_ co-incubation. The insets illustrate the contact between T_EM_ or T_CM_ with the targets. Scale bars are 20 µm. **(B)** Quantification of target cell lysis events in a time resolved death plot showing the percentage of apoptotic target cells (green), primary necrotic target cell (dark grey), secondary necrotic target cell (light grey), necrotic/lysed (grey – for semi-automated analysis) or viable cells (white) by a manual analysis. **(C-E)** Quantification of target cell lysis at 2 **(C)**, 4 **(D)** or 8 **(E)** hours by manual analysis. Data are shown as mean +SD. **(F)** Single cell values of duration/time of apoptosis for each analyzed target cell. Cells showing no signs of caspase activity at 8 hours are shown in grey. Each point reflects the Δt of apoptosis induction from a single target cell. The orange and blue lines represent the median. n=4 donors, T_EM_ 94 cells, T_CM_ 105 cells. Statistical analysis was done using a two-way ANOVA. *p<0.05; ***p<0.001; ns, no significant difference.

**Figure 5A** shows representative examples of individual NALM6 pCasper target cells which are killed after contact with sorted T_EM_ or T_CM_. For these experiments, only few T_EM_ or T_CM_ were used to favor single cell contacts. While the targets can be easily identified by their fluorescence, T_EM_ or T_CM_ are difficult to see because they are not stained. We therefore depicted an example in the insets to illustrate the contact between T_EM_ or T_CM_ with the targets. Initially all target cells are vital (orange color). The kinetic analysis revealed that T_EM_ and T_CM_ initially mainly induce apoptosis in NALM6 targets as indicated by the green color. We quantified the induction of target cell apoptosis in death plots indicated by the green areas in **Figure 5B**. T_EM_ were more efficient to induce apoptosis than T_CM_, which is evident from the initial part of the curves (**Figure 5B**) and also at the time point of 2 hours (**Figure 5C**). Over longer times, both T_EM_ and T_CM_ eliminated almost all targets by apoptosis (**Figure 5B**) as quantified at 4 hours (**Figure 5D**) and 8 hours (**Figure 5E**). The faster apoptosis induction of T_EM_ compared to T_CM_ is also evident from the median time duration of 70 min until a target cell in the T_EM_ data set was apoptotic compared to 180 min in the T_CM_ data set (**Figure 5F**).

The death plots (**Figure 5B**) also indicate that after initial apoptosis, secondary necrosis (light grey) was induced in many of the targets. Again, T_EM_ were faster to induce secondary necrosis than T_CM_ (**Figure 5B**) which is quantified for the different time points of 2, 4, and 8 hours (**Figures 5C–E**). Finally, only T_EM_ but not T_CM_, could induce primary necrosis (without preceding apoptosis) as indicated by the dark grey color (**Figures 5B–E**).Next to the perforin (and granzyme B) expression (see **Figures 3G–J**), immune synapse formation or strength could also explain the different cytotoxic efficiency of T_EM_ compared to T_CM_. Ca^2+^ influx has been used to define the initiation of degranulation and delivery of the lethal hit (i.e. 27, 28). Prior to using this approach, we first compared the amplitude of store-operated Ca^2+^ entry in T_CM_ and T_EM_. Following the typical “re-addition” protocol after thapsigargin (TG) application, we found that TCM show higher Ca^2+^ signals compared to TEM as evident from averaged Ca^2+^ traces but also from the quantitative analysis of Ca^2+^ plateau (**Supplementary Figures 2A, B**). However, when we compared Ca^2+^ signals in T_EM_ and T_CM_ following contact with Raji cells after contact, we found that they were not much different (**Supplementary Figure 2C**). Both, T_EM_ and T_CM_ responded similarly with Ca^2+^ entry once Raji cell contact was initiated. However, T_CM_ showed slightly lower Ca^2+^ signals than T_EM_, which is surprising considering the higher Ca^2+^ signals following store depletion by TG. This might be a hint that immune synapse formation in T_CM_ may be slightly less efficient than in T_EM_. Thus, immune synapse formation might in principle contribute to decreased cytotoxicity of T_CM_ compared to T_EM_, next to perforin (and granzyme B) expression. However, considering the large differences in cytotoxic efficiencies between T_EM_ and T_CM_, the slight difference in immune synapse formation between T_EM_ and T_CM_ appears unlikely to the main mechanism to mediate the different efficiencies.

### Dissecting Cytotoxic Efficiencies of T_EM_ and T_CM_

Differences of cytotoxic efficiencies between T_EM_ and T_CM_ against target cells may be caused by different mechanisms including, among others, CTL migration speed or persistence, search strategies, immune synapse formation, or different cytotoxic efficiencies during killer cell-target cell contacts. Considering that perforin, granzyme B and FasL (but not TRAIL) are expressed at higher levels in T_EM_ than T_CM_, we wanted to use the single cell cytotoxicity assay to quantify their contributions to T_EM_ and T_CM_ cytotoxicity.

To test our single cell assay and compare it to other studies, we first quantified the role of perforin, granzyme B and FasL in SEA-CTL. In comparison to untreated NALM6 pCasper target cells, blocking either perforin alone (by CMA), or perforin and granzyme B together (CMA and z-AAD-CMK in combination), or perforin and FasL together (CMA and anti-FasL antibody in combination), decreased and delayed target cell apoptosis and secondary lysis significantly (**Supplementary Figure 3A**). Quantitative analysis illustrates a very strong inhibition of necrosis and apoptosis by CMA after 2 hours (**Supplementary Figure 3B**), which is not significantly influenced by the additional use of z-AAD-CMK or anti-FasL antibody in SEA-stimulated CTL. This confirms many other studies that the perforin/granzyme B pathway is of major importance for CTL cytotoxicity (20, 21, 23, 27–30). FasL is involved but does not play a major role and granzyme B has no further impact besides its well-described role in combination with perforin (28).

**Figure 6:**
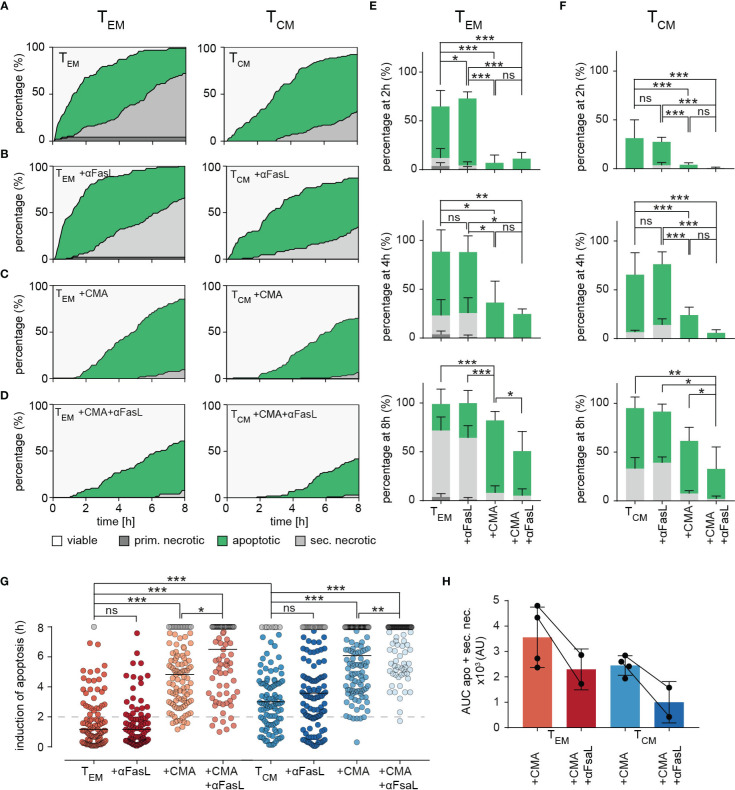
Dissecting killing mechanisms of T_EM_ and T_CM_. **(A)** To analyze target cell lysis by perforin-mediated or FasR-mediated killing mechanisms, sorted T_EM_ or T_CM_ and target cell were either un-treated as control **(A)** or treated with inhibiting FasL antibodies NOK-1 and NOK-2 (10 µg/each) **(B)**, with 50 nM CMA **(C)** and with inhibiting FasL antibodies and CMA in combination **(D)**. Effects of treatments on T_EM_ and T_CM_ are shown as representative death plots, respectively **(A-D)**. **(E, F)** Quantification of target cell lysis at 2h, 4h and 8h for T_EM_ **(E)** or T_CM_ **(F)**. **(G)** Δt of apoptosis for each analyzed target cell lysed by treated T_EM_ or T_CM_ in comparison to untreated (T_EM_ or T_CM_) control cells. **(H)** Impact of FasL blocking quantified by analysis of area under the curve (AUC). Each point reflects the Δt of apoptosis induction from a single target cell. n=4 donors, T_EM_ 94 cells, +αFasL 89 cells, +CMA 103, +CMA+αFasL (n=2 donors) 79, T_CM_ 105 cells, +αFasL 122 cells, +CMA (n=2 donors) 122 cells, +CMA+αFasL 102 cells. Statistical analysis was done using a two-way ANOVA. *p<0.05; **p<0.01; ***p<0.001; ns, no significant difference.

This analysis was now applied to T_EM_ and T_CM_. Compared to control conditions (**Figure 6A**, same plots as in **Figure 5B**), anti-FasL blocking antibody had no effect on the induction of apoptosis and subsequent secondary necrosis by T_EM_ or T_CM_ mediated cell lysis (**Figure 6B**) as also evident from the quantification of apoptosis and necrosis at 2, 4, and 8 hours for T_EM_ (**Figure 6E**) and T_CM_ (**Figure 6F**). One could argue that the antibody does not work, but this seems unlikely, as we have shown previously that it works in NK cells (24) and it also works in CTL if lytic granule exocytosis is blocked (see below).

The release of perforin from lytic granules can be blocked very well by low nanomolar concentrations of concanamycin A (CMA, (24). CMA clearly reduced target cell apoptosis and it almost eliminated primary and secondary necrosis in both T_EM_ (**Figure 6C**, quantification in **Figure 6E**) and T_CM_ (**Figure 6C**, quantification in **Figure 6F**). We conclude that perforin is required for the initial fast phase of apoptotic killing by T_EM_ and also for the slightly slower initial phase of apoptotic killing of T_CM_. The absence of perforin also eliminated necrosis. Without perforin the big difference in cytotoxic efficiency between T_EM_ and T_CM_ is diminished. Thus, the higher perforin content of T_EM_ compared to T_CM_ is involved in their increased cytotoxic efficiency.

The inhibition of perforin release by CMA in combination with anti-FasL blocking antibody significantly reduced the percentage of apoptotic target cells in the late phase of the measurement for both, T_EM_ and T_CM_ (slightly stronger in T_CM_) (**Figure 6D**, quantification in **Figures 6E, F**). Thus, if the perforin/granzyme pathway is blocked, an increased role for FasL is unmasked.

To quantify the exact time points of apoptosis induction in the target cells, every individual cell was tracked over time. The respective starting point for quantification was the first contact of a killer cell to its target cell. The exact time point of apoptosis could be quantified by loss of FRET-signals and an increase of GFP intensities (causing a switch from orange to green fluorescence). Quantification of 89 (T_EM_) or 122 (T_CM_) cells treated with CMA drastically delayed the induction of apoptosis and as expected, even more dramatically in T_EM_ (by median increased by 220 min) compared to T_CM_ (median increased by 185 min) (**Figure 6G**). Inhibition of the Fas/FasL pathway with anti-FasL blocking antibody again indicated no significant difference compared to control conditions in T_EM_ and T_CM_ subtypes. However, the combined treatment with CMA and anti-FasL blocking antibody delayed the induction of apoptosis further but to a very similar value for both subtypes, 100 min for T_EM_ and 115 min for T_CM_, respectively. Exploring the area under the curve (AUC) of the apoptotic and secondary necrotic events occurring over the whole experiment, allows the visualization and quantification of FasL-FasR interaction induced cytotoxicity (**Figure 6H**). The additional inhibition of FasL, decreases the AUC of combined apoptotic and secondary necrotic cells for T_EM_ by 35.3% and for T_CM_ by 59.2% compared to CMA alone.

Taken together these data indicate a minor role of Fas-mediated target cell lysis for both, T_EM_ and T_CM_, in case perforin/granzyme-mediated cytotoxicity is functional. After blocking perforin function and subsequent induction of apoptosis by granzyme B, apoptotic target cell death induced through FasL-FasR interaction is increasingly important for T_EM_ and T_CM_ cytotoxicity against target cells. Perforin-mediated cytotoxicity against target cells appears to be prominent in both subsets, but the compensatory role of FasL-mediated cytotoxicity against target cells appears to have a higher impact in T_CM_ compared to T_EM_.

Taken together this means that complete inhibition of perforin release by CMA and (at least) partial block of FasL-induced target cell death made both T_EM_ and T_CM_ less efficient serial killers. Nevertheless, T_EM_ were still slightly more efficient than T_CM_.

**Figure 7:**
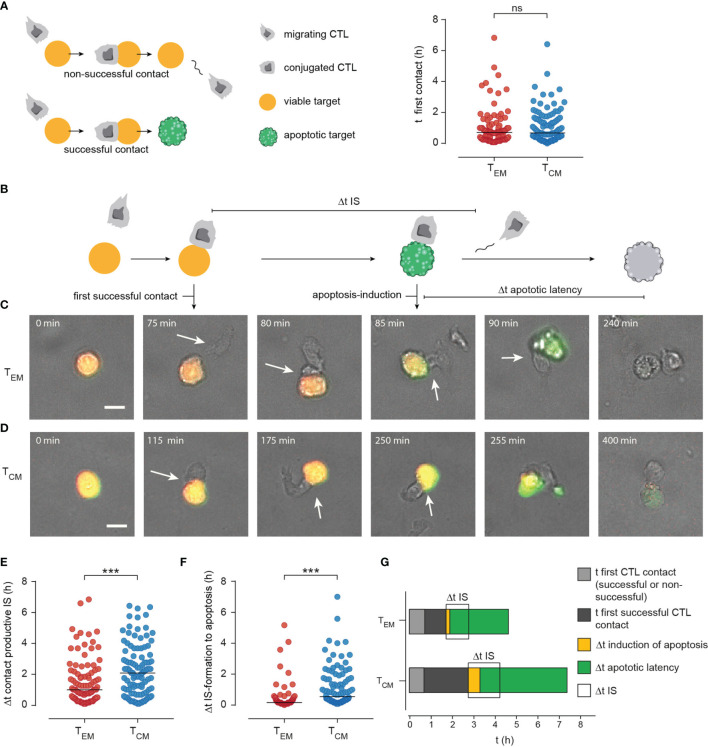
Manual tracking CTL-target cell contacts/interaction uncovers enhanced efficiency of immune synapse (IS) formation in T_EM_. **(A)** Scheme of CTL-target cell interaction: When a CTL approaches a target cell this can result in a non-productive (no induction of apoptosis or necrosis, CTL leaves the viable target after minutes – hours) or in a productive contact that results in apoptosis or necrosis of the target cell. To check if differences in target cell lysis efficiency of T_EM_ and T_CM_ are dependent on migration the time point of the first contact (whether non-productive or productive) was compared. **(B)** Scheme to illustrate the kinetics of productive CTL-target cell interaction: t of first productive contact (Δt of duration from start of the measurement until formation of a productive/lytic synapse. Time point of apoptosis induction (first signs of caspase activity detected by green fluorescence. Δt apoptotic latency: from induction of apoptosis until necrotic lysis of the cell (duration a cell was apoptotic). **(C, D)** Representative images of T_EM_ **(C)** or T_CM_ **(D)** mediated target cell lysis. Scale bars are 10 µm. **(E)** Single-cell data of time point of productive IS formation. **(F)** Single-cell data of Δt of apoptosis induction (from IS-formation to first signs of caspase activity). **(G)** Stacked bar graphs to summarize manually tracked single cell data from T_EM_ and T_CM_ mediated target cell lysis. t of first contact successful or non- successful (light grey). t of formation of the first productive contact (dark grey), Δt of apoptosis-induction (yellow), Δt of apoptotic latency (green). Δt IS: Duration of an IS between T_EM_ or T_CM_ and a target cell is shown in light grey. Durations of the phases are show as median. n=4 donors, T_EM_ 94 cells, T_CM_ 100 cells. ***p<0.001; ns, no significant difference.

It appears therefore likely that there are additional factors guiding T_EM_ efficiency in comparison to T_CM_. To test this, we quantified T_EM_ and T_CM_ target cell killing in detail. First, we quantified how long T_EM_ or T_CM_ needed to find their first target cell (**Figure 7A**). We did no distinguish whether this contact was successful or not, i.e. resulting in target cell destruction or not after T_EM_ or T_CM_ left the target. There was no difference between the time to first contact (**Figure 7A**, median T_EM_ 42.5 min to T_CM_ 40 min; n=96, 106 cells, respectively), indicating that T_EM_ and T_CM_ migration patterns or search strategies do not influence the likelihood of finding a target cell.

Next, we analyzed killing sequences in detail as displayed in **Figure 7B**. Examples for a T_EM_ and T_CM_ are shown in **Figures 7C, D**, respectively. We quantified the time to the first successful contact, i.e. the time it took a T_EM_ or T_CM_ needed to contact a target which was subsequently also killed by the CTL. The time to the first successful contact forming an IS was on average 1 hour for T_EM_ (**Figure 7C**, statistics in **7E**). This time was significantly shorter than for T_CM_ (more than 2 hours, **Figure 7D**, statistics in **7E**). In addition, during a successful response the time from IS formation to apoptosis induction was significantly shorter for T_EM_ than for T_CM_ (**Figures 7D, E**, statistics in **7F**, median 10 versus 55 min, p<0.0001) and IS duration was also shorter in case of T_EM_ which is consistent with the results previously described by Jenkins et al. The authors discovered dramatically prolonged synapse times (up to fivefold) in CTL/NK cells failing perforin and granzyme cytotoxicity, which conversely means shorter IS duration for efficient killers (31).

The differences between T_EM_ and T_CM_ are summarized in **Figure 7G**. Time to first successful contact (dark grey), duration of IS, time between IS formation and apoptosis induction in targets and also the time between apoptosis and secondary necrosis are all significantly shorter in T_EM_ than T_CM_.

In summary, whereas T_EM_ and T_CM_ need the same time to find a target cell T_CM_ need much longer than T_EM_ to establish the first contact with a subsequent successful killing. This means that T_CM_ have more failures, i.e. contacts with targets not resulting in killing. In addition, all measured time intervals (IS length, time to induce apoptosis, time to induce secondary necrosis) are shorter for T_EM_ compared to T_CM_.

## Discussion

Insights in single cell cytotoxic efficiencies of CTL subtypes are important to understand their function and to optimize adaptive T cell therapies. While the well-established and long-known ^51^Cr- release assay (32) is still considered the gold standard for detection of cytotoxicity in a CTL population, radioactivity and the lack of kinetic information are significant limitations. This led to the development of many alternative assays to quantify cytotoxicity (33–40). However, all of these are population assays, they have no single cell resolution and do not discriminate between apoptosis and necrosis. To differentiate apoptosis from necrosis or alternate cell death, Waterhouse et al. used time-lapse microscopy for single cell analysis. They analyzed CTL induced target cell death by adding propidium iodide (PI) and annexin V-FLUOS to the culture dish up to 3.0 hours (29). However, a disadvantage of using these two dyes might be the different fluorescence intensities. In another study, Lopez et al. used time-lapse microscopy to follow OT-I CTL cytotoxicity against MC57-OVA257 targets in the presence of 100 µM PI (28). Interestingly, they visualized killing of CTL by a combined strategy following the Ca^2+^ signals in effector cells and the addition of PI (27) and could identify distinct Ca^2+^ signals in human compared to murine CTL.

To quantify T_EM_ and T_CM_ cytotoxicity, a FRET-based caspase-dependent cytotoxicity assay was developed for CTL, which was previously shown to discriminate between apoptosis and necrosis at a single cell level (24, 41). The pCasper construct used here for quantification of caspase/protease is not the only one available, and others have been used to detect cell apoptosis by luciferase activity, quenching, or change in the fluorophore localization (21, 42, 43). However, none of these have been used for the detection of single CTL cytotoxicity.

Our study combines population measurements and the FRET-based single cell assay which allows the precise quantification of T_EM_ and T_CM_ cytotoxic modes and efficiencies. All experiments revealed that the cytotoxic efficiency against target cells is higher for T_EM_ than for T_CM_. This could in principle be the result of several different mechanisms:

i) Faster and/or more persistent migration of T_EM_ comparted to T_CM_. This is unlikely as T_EM_ and T_CM_ require similar times to contact their first respective target.
ii) Different effectivity of cytotoxic mechanisms. Several groups (20, 21, 27–30) including our own (23) have previously reported that perforin and granzymes are very important in CTL for the initial lysis phase. T_EM_ lyse targets faster than T_CM_. Thus, differential perforin and/or granzyme expression or release could be responsible for the differences. Since perforin is higher expressed in T_EM_ than T_CM_, since we found a correlation between perforin expression and lysis rates for the different CTL subtypes, and since pharmacological inhibition of perforin release decrease lysis of target cells, we hypothesize that perforin (together with granzymes) has an important role to explain cytotoxic differences between T_EM_ and T_CM_. However, the perforin expression level alone does not fully explain the lytic differences between TEM and TCM, because cytotoxicity is still different when perforin release is fully blocked. FasL/FasR induced apoptosis appears not be different between T_EM_ and T_CM_, and expression levels of FasL were similar between T_EM_ and T_CM_. Thus, other death pathways like TRAIL-dependent cytotoxicity could contribute, however we did not observe any differences regarding TRAIL expression (**Figures 3K, L**).
iii) Efficiency of immune synapse formation could be different between T_EM_ and T_CM_. We analyzed the efficiency of immune synapse formation by quantifying Ca^2+^ signals as done previously by Lopez et al. (28) (**Supplementary Figure 2**). Considering the smaller Ca^2+^ signals in T_CM_ compared to T_EM_ following contact with their targets, it is possible that differences in immune synapse formation contribute to differences between T_EM_ and T_CM_ efficiency.

In summary, we conclude that perforin (and granzyme) expression and potentially immune synapse formation are involved in the increased cytotoxicity of T_EM_ compared to T_CM_. Considering the small residual cytotoxicity after perforin/granzyme and FasL inhibition, further mechanisms like TRAIL-dependent cytotoxicity are probably important in both, T_EM_ and T_CM_.

A detailed understanding of CD8^+^ T cell subtype development, composition and killing mechanisms is of great importance to optimize future adaptive cell therapies such as the use of CAR T cells. To date, molecular signatures, the signaling pathways of cytotoxic mechanisms and the cytotoxic receptor repertoire of death receptor or perforin-mediated target cell lysis of different CD8^+^ T cell subtypes have been comprehensively investigated, frequently flow cytometry or more recently mass cytometry based methods have been used (44–46). In contrast, the functional *in vitro* analysis of the cytotoxicity of human CTL subtypes is not on a comparable level, especially considering the single cell level. In this manuscript we have elucidated the cytotoxic potential of human CD8^+^ T cell memory subtypes T_EM_ and T_CM_ and the relevance of their cytotoxic mechanisms. Staphylococcal enterotoxin A (SEA)-stimulation of CD8^+^ T cells, which permits a subsequent analysis of the cytotoxicity against of SEA-loaded target cell (23, 47, 48), preferentially led to the differentiation into a TCM subtype. Nevertheless, the size of the T_EM_ and T_N_ populations was still large enough to have clearly defined subtypes available for further analysis. Only the terminally differentiated T_EMRA_ could not be included in further analysis which is a limitation of SEA stimulation.

From a clinical perspective these data suggest a potential advantage of selecting CTL subtypes for virus specific T cells, e.g. CMV-specific, or for the generation of CAR T cells. While there is only a 2.8-fold difference of granular perforin between T_EM_ and T_CM_, the strong kinetic difference of T_EM_ compared to T_CM_ cytotoxicity may favor the use of T_EM_. However, a limiting factor is certainly to problem that patient-derived T cells are often poor in quantity and quality due to many previous therapies including purine analogues among others, so that a pre-selection of the initial T cell subset requires more efficient transduction and expansion protocols. Interestingly, superior results have been achieved with CAR T cells using fixed CD4 to CD8 ratios (12, 13). A fixed composition of different T-cell subsets would be more feasible for off-the shelf products. A memory-derived (T_CM_ and T_SCM_) CD19 CAR T cell subtype has also been shown to be beneficial due to its sustained proliferative potential and specific cytotoxicity (49). Safety and feasibility of T_CM_-derived CD19 CART-cell therapy was demonstrated for treatment of poor-risk NHL patients undergoing autologous HSCT (hematopoietic stem cell transplantation) (50). In addition, the selection of the costimulatory domain also influences differentiation into specific subtypes. While CD28 as a co-receptor has tended to lead to the development of an T_EM_ subtype, 4-1BB favors the T_CM_ subtype (51).

Our data suggest that CTL, and thus further developed CAR T cells might have the ability to kill target cells harboring defective apoptosis mechanisms by direct cytolysis, which is clinically relevant. For example, deletions in the short arm of chromosome 17 (del17p) includes the coding region for the tumor suppressor p53 (TP53) associated with a poor diagnosis e.g. in CLL (chronic lymphocytic leukemia) (52).

An important issue for the success of CAR T cell therapy remains the understanding and investigation of resistance mechanisms. Poor persistence of CAR T cells as well as antigen loss or modulation of antigen on tumor cells lead to shortened remission (53). The data of this work suggest that resistance mechanisms against perforin might be of particular interest in this context. For CTL, high lipid order and exposed phosphatidylserine has already been shown to be protective against perforin-induced cell death (54). To consider strengthening the effect of compensatory mechanisms such as the death domain ligands FasL and TRAIL therapeutically, for example by BCL2 inhibitors.

## Materials and Methods

### Ethical Approval

Research with human PBMC has been approved by the local ethic committee (84/15; Prof. Dr. Rettig-Stürmer). We got the leukocyte reduction system (LRS) chambers, a by-product of platelet collection from healthy blood donors, from the local blood bank (Institute of Clinical Hemostaseology and Transfusion Medicine, Saarland University Medical Center). All blood donors provided written consent to use their blood for research purposes.

### Reagents

Anti-human CD178 was obtained from BD Biosciences, anti-human CD95 Apo 1-1 was from Enzo Life Sciences. Flow cytometry antibodies (for details see *Flow Cytometry*) and apoptosis inducing TRAIL-Antibody were purchased from Biolegend. IL-7 was from Peprotech, IL-12 and IL-15 were from Miltenyi. Nucleofector Kit V was from Lonza. The original vector pCasper-GR (#FP971) was from Evrogen. BSA, fibronectin and staurosporine from Streptomyces sp. were from Sigma-Aldrich. The Dynabeads CD8 Positive Isolation Kit, fetal bovine serum (FBS) (EU-approved), IL-2, G418, PBS, RPMI-1640, AIM-V medium, penicillin/streptomycin (P/S) and were from ThermoFisher Scientific. Concanamycin A (CMA) was from Santa Cruz Biotechnology. All other chemicals and reagents not specifically mentioned were from VWR, ThermoFisher Scientific or Sigma-Aldrich.

### Cells Lines and Primary Human CD8^+^ T Cells

Raji cells (ATCC, #CCL-86) and NALM6 cells (DSMZ, #ACC128) were cultured in RPMI-1640 (ThermoFisher Scientific) supplemented with 10% FBS and 1% Penicillin/Streptomycin at 37°C and 5% CO_2_. Human peripheral blood mononuclear cells (PBMCs) were isolated from the leukoreduction system chamber (LRSC) as a by-product from healthy thrombocyte donation as described previously (55).

PBMCs were stimulated with staphylococcal enterotoxin A (SEA; 0.5 μg/ml) in a 24 well plate (density of 1–1.5 × 10^8^ cells/ml; 37°C for 1 h). PBMCs were resuspended at a density of 2–4×10^6^ cells/ml in AIM-V medium supplemented with 10% FBS and 100 U/ml recombinant human IL-2. 5 days after stimulation, SEA-specific CTL were positively isolated using a Dynabeads CD8 Positive Isolation Kit (ThermoFisher Scientific).

Purified CD8^+^ cells were cultured in AIMV medium supplemented with 10% FBS and 100 U/ml recombinant human IL-2 (ThermoFisher Scientific) and used for sorting of CD8^+^ T cell subsets and for further experiments three days after isolation.

### Generation of Stable NALM6 pCasper Cell Line

The sequence encoding for Casper3-GR was amplified from pCasper3-GR (Evrogen # FP971) with the following primers introducing an XhoI recognition site at both ends of the amplicon. Forward primer: 5’-CTCGAGGCCACCATGGTGAGCGAG-3’, reverse primer: rev 5’-GACGAGCTGTACCGCT GACTCG AG-3’. The amplicon was subcloned into the XhoI site of a previously modified pGK-Puro-MO70 vector backbone were CITE-EGFP sequence was deleted (56). The generated plasmid encoded puromycin resistance gene and was used for selecting transfected NALM6 cells. 1 x 10^6^ NALM6 target cells were transfected with 1 µg plasmid using the Nucleofector 4D (SF cell line Kit, program CV-104, Lonza) following the manufacturer’s instruction. After 48 hours, selection started with 0.2 µl/ml puromycin (1 mg/ml stock solution). To enrich the pCasper GFP^+^/RFR^+^ population for clonal selection, GFP- and RFP-positive cells were sorted on a FACSARIAIII sorter and plated as 1 cell/per well in 96 well plates. GFP- and RFP-positive clones were selected by the ImageXpress Micro XLS screening image analysis system (Molecular Devices), expanded and frozen in 90%FBS/10%DMSO for further use.

### Flow Cytometry

The frequency of CD8^+^ T cell subpopulations was examined by intra- and extracellular stainings. 5x10^5^ cells were washed twice in PBS/0.5% BSA, stained with PerCP-conjugated anti-human CD3 (SK7, Biolegend), FITC-conjugated anti-human CD8 (SK1, Biolegend), PE/Cy7-conjugated anti-human CD45RA (Hl100), Biolegend) or CD45RO (UCHL-1, Biolegend), PerCp/Cy5.5-conjugated anti-human CD62L (DREG-56, Biolegend), Alexa Fluor-647-conjugated anti-human CD197 (150503, BD Biosciences), PE-conjugated anti-human CD253 (308205, Biolegend) antibodies in PBS/0.5% BSA, washed twice and recorded on a BD FACSVerse flow cytometer (BD Biosciences). For intracellular stainings, cells were fixated in 4% PFA in PBS for 20 min, washed in PBS/0.5% BSA, permeabilized for 10 min in PBS/0.1% saponin and stained with FITC-conjugated anti-human Perforin (δG9, Biolegend) in 0.1% PBS/0.1% saponin. After staining cells were washed and acquired on a FACSVerse flow cytometer.

For analysis of degranulation and staining of the death receptors FasL and TRAIL, 3x10^5^ SEA-pulsed target cells or target cells without SEA as control were seeded in wells of a 96-well plate. 6x10^5^ SEA-CTL were added to each well and cells were co-incubated in the presence of Brilliant Violet 421-conjugated anti-human CD107a antibody. After 4 h cells were collected on ice, washed (4°C) and a surface staining of CD45RA, CCR7, PE-conjugated anti-human CD178 (NOK-1, Biolegend) or PE-conjugated anti-human CD253 (RIK-2, Biolegend) was performed, cells were washed before analysis on a FACSVerse flow cytometer (BD Biosciences).

T_EM_ or T_CM_ were generated from human PBMC (peripheral blood mononuclear cell) using a widely accepted protocol for polyclonal activation of T cells by staphylococcal enterotoxin A (SEA) (47, 57) that importantly allows the subsequent analysis of SEA-dependent cytotoxicity (39, 48). For sorting 4x10^7^ SEA-CTL were stained with anti-CD62L and anti-CD45RO in PBS + 0.5% BSA, washed, strained (30 µm) and sorted on a BD FACSAria III (70 µm nozzle). Cells were collected in PBS/0.5% BSA/25% FBS, washed and cultured in AIM-V + FBS + 100 ng/ml IL-2. T_EM_ received 25 ng/ml IL-2, 5 ng/ml IL-12, 10 ng/ml IL-15, T_CM_ received 25 ng/ml IL-2, 5 ng/ml IL-7 and 10 ng/ml IL-15. Cells were measured on a BD FACSVerse flow cytometer and data analysis was performed using FlowJo.

### Real-Time Killing Assay

To quantify the cytotoxicity of SEA-stimulated CD8^+^ T cells against SEA-pulsed Raji or NALM6 target cells we used a time resolved, real-time killing assay that was carried out essentially as previously described (39). Briefly, target cells (Raji or NALM6) were pulsed with 1 µg/ml SEA in AIM-V medium at 37°C and 5% CO_2_ for 30 min. Next, cells were loaded with 500 nM Calcein-AM in AIM-V medium supplemented with 10 mM HEPES for 15 min at room temperature. After one washing step, 2.5 x 10^4^ target cells per well were pipetted into a 96-well black plate with clear-bottom (#7342480, VWR). After a short rest (15 min) to let target cells settle down, effector cells were added at the indicated effector to target ratio (E:T) and target cell lysis was measured in a Genios Pro (Tecan) reader using bottom reading function over 4 h at 37°C every 10 minutes. Maximal lysis rates were calculated as the maximum increase between two subsequent measured points (10 minutes).

### Single Cell Imaging and Target Cell Death Analysis

Single cell killing assay measurements were run on an ImageXpress Micro XLS screening image analysis system (Molecular Devices). The detection of apoptotic and necrotic target cells with the apoptosis sensor Casper-GR construct was carried out as described before (24). Briefly, NALM6 pCasper target cells were pulsed with 1 µg/ml SEA in AIM-V medium at 37°C and 5% CO_2_ for 30 min, washed taken up in 100 µl of phenol-free RPMI-1640/10% FBS and seeded into fibronectin coated wells (coating 30 min, 50 µl of 0.1 mg/ml fibronectin per well) of a 96-well plate (1x10^4^ targets per well). After a short rest (30 min) 2x10^4^ effector cells in 100 µl of phenol-free RPMI-1640/10% FBS were added per well and the measurement was started. Over 8h bright field, GFP- and FRET-signals were acquired with a 20x objective from 1-4 positions per well at a 5 min interval. Objects were excited *via* Spectra X LED illumination (Lumencor) using LED 470/24 for GFP. The filter sets for FRET were 472/30 nm for excitation and 520/35 nm for emission to measure GFP and 641/75 nm to measure RFP. A Nikon Super Fluor objective (20x/NA 0.75) was used.

### Semi-Automated Target Cell Death Analysis

A semi-automated analysis to quantify results shown in **Supplementary Figure 3** was performed in the following way. The spot detection algorithm of the software Imaris (Bitplane, V8.1.2) was used to identify target cells based on their average size and GFP-fluorescence signal. To avoid potential errors of a completely automated tracking caused by collision or cell contacts the detected spots were not connected over time. The number of target cells and their fluorescence signals can only be quantified for each single time point separately. Due to this reason, it is not possible to determine if a necrotic cell death was due to primary or secondary necrosis (because the state of a cell in the previous time points is unknown). GFP- and FRET fluorescence values were exported into Excel and analyzed as described in the manual tracking section above. Necrotic target cells lose their fluorescence signal and cannot be detected by the spot detection algorithm. We set the total number of detected spots in the first pictures (maximum of total detected spots, before CTL-target cell contact set) to 100% and used the difference in number of detected spots at later time points to estimate the proportion of undetected necrotic target cells. The proportion of apoptotic, primary necrotic or secondary necrotic cells at any given time point was calculated and displayed in a color-coded death plot diagram over time (24).

### Treatment With Inhibitory Substances

To inhibit perforin-mediated target cell lysis effector cells were preincubated for 2 h in phenol-free RPMI-1640/10% FBS supplemented with 50 nM concanamycin A (CMA) and 50 nM CMA was also present during the measurements. 1x10^4^ targets per well were re-suspended in 100 µl phenol-free RPMI-1640/10% FBS/50 nM CMA. Effector cells were added in 100 µl of phenol-free RPMI-1640/10% FBS/50 nM CMA after preincubation.

To inhibit granzyme B-mediated target cell lysis, effector cells were preincubated for 2 h in phenol-free RPMI-1640/10% FBS supplemented with 10 µM z-AAD-CMK and 10 µM z-AAD-CMK was also present during the measurement. To this end 1x10^4^ targets per well were submitted in 100 µl of phenol-free RPMI-1640/10% FBS/10 µM z-AAD-CMK. Effector cells were added in 100 µl of phenol-free RPMI-1640/10% FBS/10 µM z-AAD-CMK after preincubation.

To block FasL- and TRAIL-mediated target cell lysis inhibitory anti-human antibodies were used to block the interaction of FasL or TRAIL with their respective apoptosis inducing receptors. To this end 1x10^4^ targets per well and 2x10^4^ effector cells per well were each preincubated for 2 h in 10 µg/ml anti-human CD178 antibodies (NOK-1 and NOK-2, BD Biosciences) or 5 µg/ml anti-human TRAIL antibody (RIK-2, Biolegend). After 2 h of preincubation effector cells were added to target cells.

### Manual Target Cell Tracking and Cell Death Analysis

In principle the manual analysis was carried out as described before (24). Briefly: Roughly 25 target cells were chosen randomly for each recorded position and tracked with Speckle Tracker J. The center of each target cell was marked at each time point manually. X,y positions and GFP- and FRET fluorescence signals were exported for every tracked cell with the speckle tracker intensity trajectories plugin. Data were imported into Excel to perform normalization to the starting value of a target cell and calculate the donor ratio for each cell. The GFP- and FRET-signal as well as the donor ratio were analyzed to determine the fate of a cell, viable (donor ratio > 0.75), apoptotic (donor ratio < 0.75) or necrotic (loss of both fluorescence signals, threshold FRET-value 10% above background signal; FRET< 150 units). This was used to identify the status of each tracked target cell at every time point. A cell being apoptotic prior to necrosis was defined as secondary necrosis. A necrotic event without a rise of the GFP-signal and an accompanying drop of the donor ratio was defined as primary necrosis. The sum of all detected cells per condition was set to 100% and the proportion of apoptotic, primary necrotic or secondary necrotic cells at any given time point was calculated and displayed in a color-coded death plot over time.

### Image Analysis of Stable NALM6 pCasper Target Cell Line

Images were analyzed as described in (24). Briefly, background correction *via* rolling ball, F/F0 normalization and donor ratio calculation were performed in ImageJ (NIH, version1.51d) and Microsoft Excel (Microsoft Office 2016). Following rolling ball background subtraction in ImageJ measurements were tracked manually with Speckle TrackerJ plugin for ImageJ.

### Single Cell Ca^2+^ Imaging

Ca^2+^ imaging was essentially as described in Schwarz et al. (58). Briefly, 3 x 10^5^ T_EM_ or T_CM_ were loaded at 22–23 °C for 20 min with 1 µM fura-2/AM (Thermo Fisher Scientific) in medium, washed with fresh medium, stored at room temperature for 10 min, and immediately used. For Ca^2+^ re-addition experiments, T_EM_ or T_CM_ were allowed to adhere to poly-L-ornithine-coated (0.1 mg/mL) glass coverslip, which were assembled in self-build chambers on the stage of an Olympus IX 70 microscope. Measurements were carried out at 22–23 °C. A 20x objective (UApo/340, N.A. 0.75) was used, and cells were alternately illuminated at 340 and 380 nm with a Polychrome IV or V Monochromator (TILL Photonics) using SP 410 as excitation filter and DCLP 410 as dichroic mirror. The fluorescence emissions at >440 nm (LP 440) were captured with a CCD camera (TILL Imago), digitized, and analyzed using TILL Vision software. Ratio images and infrared images were recorded at intervals of 5 s. Background-corrected 340 nm/380 nm ratios were analyzed for each cell marked by “regions of interest” (ROIs) covering the whole cell. For analyzing Ca^2+^ signals of T_EM_-Raji or T_CM_-Raji contacts, the following modifications were made. Fura/AM-loaded T_EM_ or T_CM_ were settled down on Raji cells, already adhered to fibronectin-coated coverslips to allow T_EM_ or T_CM_ migration. All T_EM_-Raji or T_CM_-Raji contacts were analyzed. Ca^2+^ traces were aligned to the beginning of contact between T_EM_ or T_CM_ and Raji cells as indicated in the respective figure.

### RNA Isolation and Quantitative RT-PCR

All molecular biology experiments were done as described before (59). Briefly, total RNA was isolated from 1.0 to 1.5 × 10^6^ CD8^+^ T-cells using TRIzol reagent (ThermoFisher Scientific) including 1 μl Glycogen (5 μg/μl, ThermoFisher Scientific). 0.8 μg total RNA was reverse transcribed to cDNA and 0.5 μl of cDNA was used for quantitative real time polymerase chain reaction (qRT-PCR). QRT-PCR was carried out in a CFX96™ Real-Time SystemC1000™Thermal Cycler (Biorad CFXManager). Primers used in this study: RNAPol and TBP as reference genes (59), FASL: forw 5′ GCACACAGC ATCATCTTT 3′, rev 5′ CAAGATTGACCCCGGAAGTA 3′; perforin (NM_005041 and NM_001083116) forw 5′ ACTCACAGGCAGCCAACTTT 3′, rev 5′ CTCTTGAAGTCAGGGTGCAG 3′; granzyme B, forw 5′ GAGACGACTTCGTGCTGACA 3′ and rev 5′ TCTGGGCCTTGTTGCTAGGTA 3′. QuantiTect primers for IFNγ, TNFα and TRAIL were purchased from Qiagen (QT00000525, QT00029162, QT00079212).

### Data and Statistical Analysis

Data were tested for significance using one-way Anova, Mann-Whitney-Test, Kruskall-Wallis-Test with a Dunn´s multiple comparisons post-test: * p<0.05; ** p<0.01; *** p<0.001; ns, no significant difference as stated in the figure legends. Data analyses were performed using Microsoft Excel 2016, FlowJo, Imaris (Bitplane, V8.1.2), ImageJ, Igor Pro (WaveMetrics) and GraphPad Prism 7 software.

## Data Availability Statement

The raw data supporting the conclusions of this article will be made available by the authors, without undue reservation.

## Ethics Statement

The studies involving human participants were reviewed and approved by Ethik-Kommission bei der Ärztekammer des Saarlandes, Saarbrücken 84/15; Prof. Dr. Rettig-Stürmer. The patients/participants provided their written informed consent to participate in this study.

## Author Contributions

AK carried out the experiments, performed the analysis and was involved in writing the manuscript. GS, DA, and JR performed experiments. LT helped with writing the manuscript. MH contributed to planning experiments and writing of the manuscript. ES supervised the project and wrote the manuscript. All authors contributed to the article and approved the submitted version.

## Funding

This work was supported by the Deutsche Forschungsgemeinschaft (DFG, the collaborative research centers SFB 1027 (project A11 to MH), SFB 894 (project A1 to MH), DFG, State Major Instrumentation, GZ: INST256/429-1 FUGB (Molecular Devices, High content Screening System) and GZ: INST 256/423-1 FUGG (BD Biosciences, FACSVerse), and by the Bundesministerium für Bildung und Forschung (BMBF, grant 031LO133 to MH).

## Conflict of Interest

The authors declare that the research was conducted in the absence of any commercial or financial relationships that could be construed as a potential conflict of interest.

## Publisher’s Note

All claims expressed in this article are solely those of the authors and do not necessarily represent those of their affiliated organizations, or those of the publisher, the editors and the reviewers. Any product that may be evaluated in this article, or claim that may be made by its manufacturer, is not guaranteed or endorsed by the publisher.

## Abbreviations

T_EM_, CD8^+^ effector memory; T_CM_, CD8^+^ central memory; CTL, CD8^+^ T cells; LRS chambers, leukocyte reduction system chambers.
