# Supplementary material for: Cytotoxic Efficiency of Human CD8+ T Cell Memory Subtypes
Source: Front Immunol. 2022 Apr 13;13:838484. doi: 10.3389/fimmu.2022.838484 (PMC9043813; doi:10.3389/fimmu.2022.838484)
Supplement: Supplementary file 1 [file DataSheet_1.pdf]

Cytotoxic efficiency of human CD8⁺ T cell memory subtypes

Arne Knörck, Gertrud Schäfer, Dalia Alansary, Josephine Richter, Lorenz Thurner, Markus Hoth, Eva C. Schwarz

Supplementary Figures 1-3

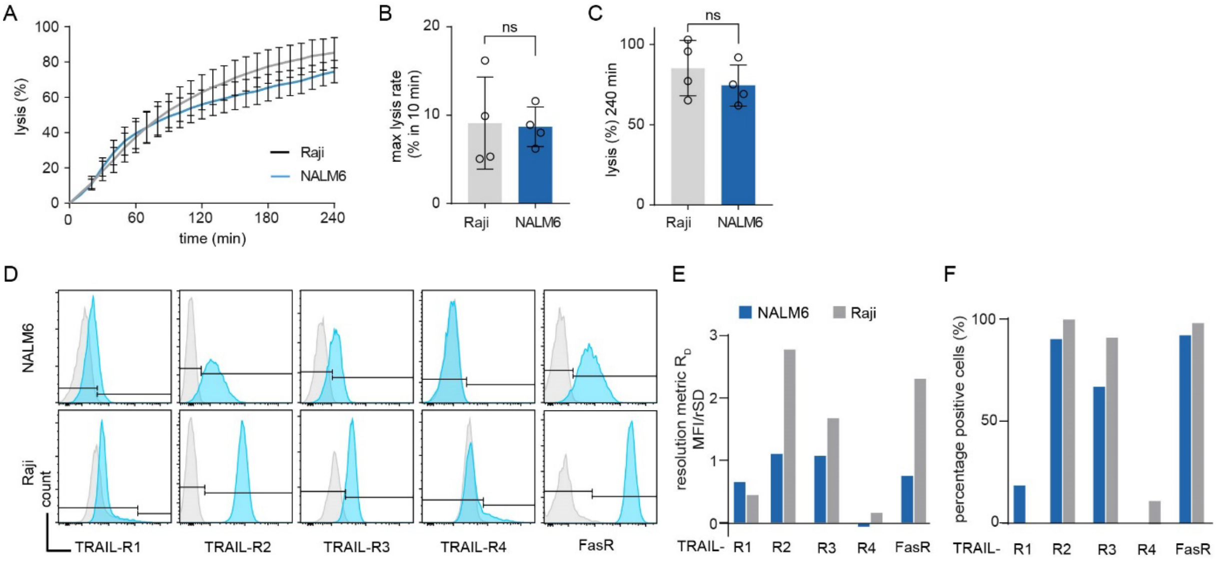

Supplementary Figure 1: Comparison of Raji- and NALM6 target cell systems: SEA-mediated cytotoxicity and expression of FasR and TRAIL receptors 1-4. A) Cytotoxicity of SEA-CTL against target cells Raji (grey) or NALM6 (blue) was analyzed by calcein-based real-time killing assay over 240 min. Effector to target ratio (E:T) was, n=4 donors, data are shown as mean +SD. Analysis of the maximal lysis rate (B) and end point target lysis after 240 min (C). D-F) FasR and TRAIL receptor 1-4 expression of Raji and NALM6 target cells was analyzed by flow cytometry (n=1). Quantification was done by determining the resolution metric (RD) value (MFI_{probe} – $MFI_{IgGcontrol}$/(rSD_{probe} + $rSD_{IgGcontrol}$) (E). F) Proportion of TRAIL receptor 1-4 (R1-R4) and FasR positive cells. ns, no significant difference.

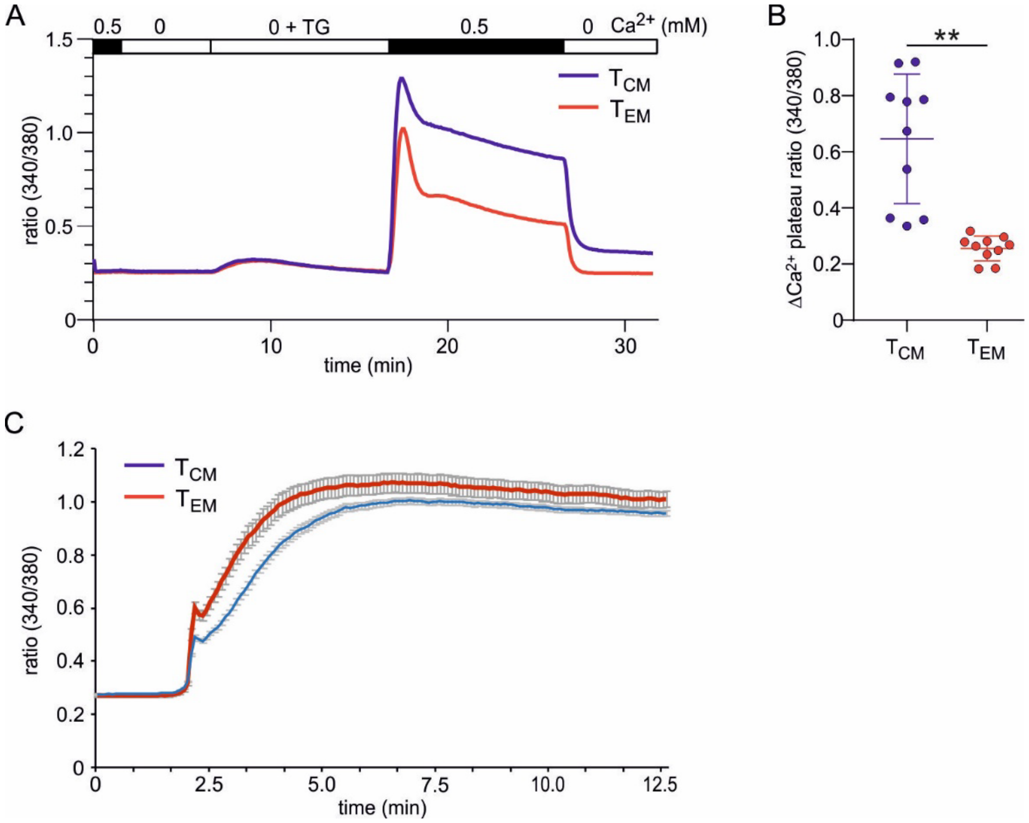

Supplementary Figure 2: Ca^{2+} signals in T_{CM} and T_{EM}. A) Mean Ca^{2+} traces of T_{CM} and T_{EM} subtypes after store depletion with thapsigargin (TG) using a Ca^{2+} re-addition protocol. B) Ca^{2+} plateau analyzed from 6 different donors (for each subtype) measured once or twice (n=10, 66-255 cells each measurement). Data are shown as mean±SD. C) Synchronized Ca^{2+} influx of T_{CM} and T_{EM} after target cell contact (Raji cells). Data are shown as mean±SEM (n=1 donor, 67 (T_{EM}) and 106 (T_{CM}) cells). ** $p<0.01$.

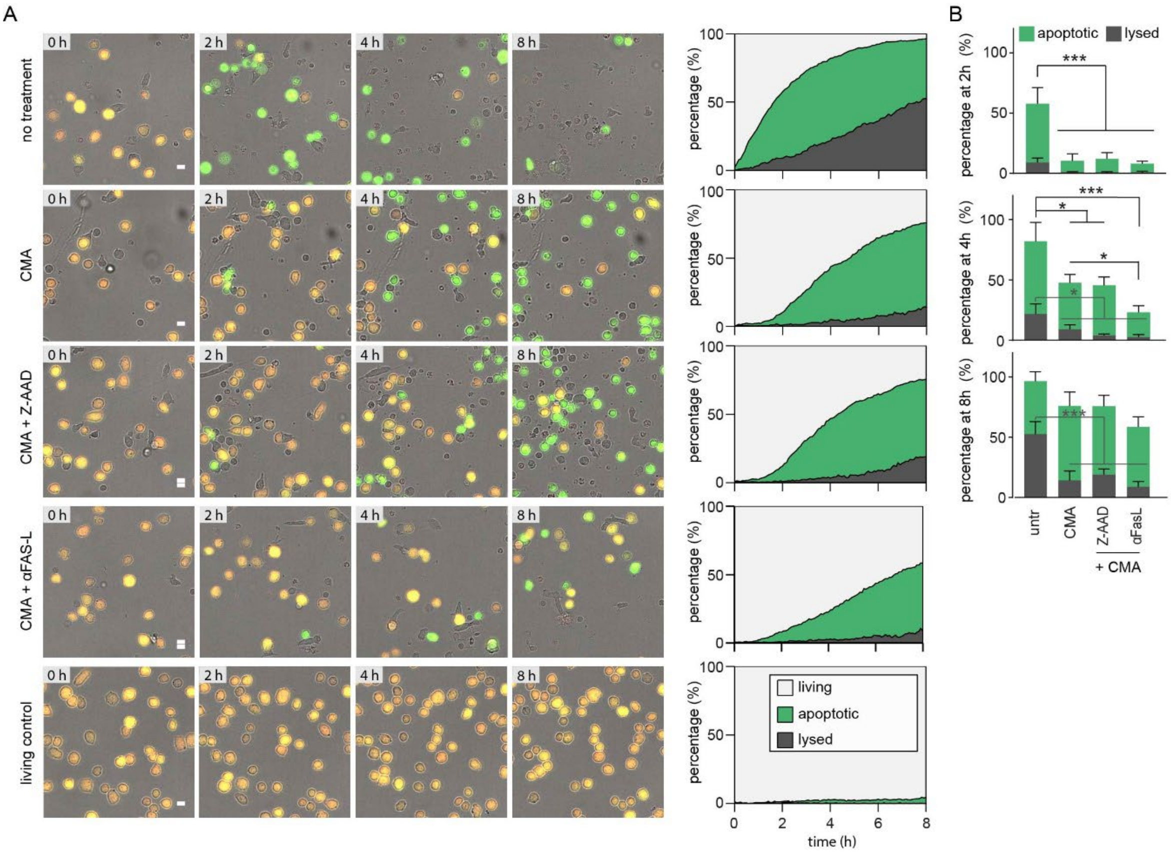

Supplementary Figure 3: Dissecting killing mechanisms by blocking different killing pathways. A) SEA-CTL mediated lysis of SEA-pulsed NALM6 pCasper-GR cells over 8h is quantified by semi-automated analysis and shown as a death plot. From top to bottom: no treatment (n=916 cells), effect of perforin inhibition by 50 nM CMA (n=1180 cells), effect of combined inhibition of perforin and granzyme B by 50 nM CMA + 20 µM z-AAD-CMK (n=1055 cells), effect of combined inhibition of 50 nM CMA + anti-FasL (NOK-1 and NOK2, 10 µg/ml each) (n=1158 cells), NALM6 pCasper target cells without CTL-SEA treated with all inhibitory treatments (living control) n=585. B) Quantification showing the percentage of apoptotic and necrotic/dead target cell at 2h, 4h and 8h. Statistical analysis was done using a two-way ANOVA test. Scale bars are 10 µm. * $p<0.05$; *** $p>0.001$.
